# A Comprehensive Review on the Influence of Menstrual Cycle and Pregnancy on Epileptic Seizures

**DOI:** 10.7759/cureus.80387

**Published:** 2025-03-11

**Authors:** Hugh Kolomar, Alaa Osman, Lizeth Valeria Arias Blanco, Fay Ali Alotaibi, Irlanda Lince Flores del Valle, Saacha F Mohammed, Shreya Singh, Farah Algitagi, Esaúl Marroquín León

**Affiliations:** 1 Immunology, Nis University, Nis, SRB; 2 Medicine, Lebanese University, Beirut, LBN; 3 Medicine, Fundación Universitaria Sanitas, Bogota, COL; 4 Neurology, King Fahd Military Medical Complex, Dhahran, SAU; 5 Medicine, National Autonomous University of Mexico, Pachuca, MEX; 6 Medicine, Trinity College Dublin, Dublin, IRL; 7 Medicine, Ternopil National Medical University, Ternopil, UKR; 8 Medicine, Hashemite University, Zarqa, JOR; 9 Medicine, Universidad Westhill, Mexico City, MEX

**Keywords:** catamenial epilepsy, epileptic seizures, menstrual cycle, pregnancy, progesterone

## Abstract

This narrative review aims to assess the influence of hormonal balance during the menstrual cycle and pregnancy on the severity of epileptic seizures. Hormonal fluctuations, particularly in estrogen and progesterone levels, significantly influence neuronal excitability and seizure thresholds, with estrogen generally enhancing excitatory activity and progesterone providing a stabilizing effect. These hormonal variations are particularly evident in conditions like catamenial epilepsy, where seizure frequency corresponds to specific phases of the menstrual cycle. Pregnancy adds further complexity, as shifts in hormonal dominance can lead to variable effects on seizure patterns. Understanding these interactions is crucial for developing tailored management strategies to improve outcomes for women with epilepsy during their reproductive years. This review consolidates existing knowledge and highlights areas where further research is needed to address clinical challenges and optimize care.

## Introduction and background

Epilepsy is a common chronic neurological disorder marked by repeated and unprovoked seizures caused by abnormal electrical activity in the brain [1]. Seizures occur when large groups of nerve cells (neurons) fire simultaneously, disrupting normal brain function [2]. It affects both sexes and roughly 1% of the global population, with a high prevalence in reproductive-aged women (7.62 cases per 1,000 persons) [3-5]. In the United States alone, approximately 1.7 million women live with epilepsy [6].

Managing epilepsy in women presents unique challenges due to the influence of hormonal fluctuations. During pregnancy, approximately 20% to 35% of epileptic women have an increase in seizure frequency [7]. Research shows that seizure frequency increases in approximately 20% to 35% of pregnant women with epilepsy [7]. Additionally, some women experience changes in seizure patterns linked to their menstrual cycle, a phenomenon known as catamenial epilepsy, which affects an estimated 10% to 70% of women with epilepsy [6,8,9]. In addition, females are up to three times more likely to develop any mood disorder in comparison to males [2]. These gender differences are due to hormonal changes, specifically fluctuations in estrogen and progesterone levels, which can affect neuronal excitability and seizure thresholds.

The interaction between hormones and seizure activity is a critical area of study. Hormones not only influence brain excitability but are also impacted by seizures, leading to disruptions in reproductive endocrine function, such as menstrual abnormalities, including irregular cycles, oligomenorrhea, polymenorrhea, or amenorrhea. This bidirectional relationship highlights the importance of understanding the role of hormones in epilepsy, especially in the context of the menstrual cycle and pregnancy [10,11].

Seizure fluctuations for women are primarily modulated by the two primary reproductive hormones that play opposing roles: the pro-convulsant estrogen and the anticonvulsant progesterone and its metabolites [12]. Estrogens, particularly estradiol (E2), increase seizure susceptibility by their excitatory effects on the brain. E2 enhances neuronal excitability by increasing dendritic spine density and N-methyl-D-aspartate (NMDA) receptor activity by acting as a posttranscriptional modulator in the excitatory CA1 pyramidal neurons in the hippocampus and by reducing inhibitory GABAergic neurotransmission over time. Conversely, progesterone, primarily through its metabolite allopregnanolone (AP), a potent positive allosteric modulator of the GABA receptor, exerts anticonvulsant effects. These effects are mediated by potentiating GABAergic activity via GABA receptor modulation [6, 12].

The hormonal changes that occur during pregnancy further complicate epilepsy management. Unlike estradiol, which increases seizure susceptibility, another form of estrogen, estriol, becomes dominant during pregnancy and may have a different effect on seizure activity [10]. At the same time, progesterone levels steadily rise. Understanding how these hormonal shifts influence seizures during pregnancy remains an area of active research.

This review aims to explore how hormonal fluctuations during the menstrual cycle and pregnancy influence epilepsy. By consolidating current knowledge, we seek to clarify existing research gaps, improve clinical awareness, and guide management strategies to enhance health outcomes for women with epilepsy during reproductive phases.

## Review

Hormonal modulation and menstrual cycle in epilepsy: catamenial epilepsy 

Catamenial epilepsy is defined as an increase in the frequency of epileptic seizures related to specific phases of the menstrual cycle in women with epilepsy. It is characterized by increased seizure frequency during the premenstrual and periovulatory periods in the ovulatory cycles and the luteal phase in the anovulatory cycles [13].

Studies have found that menstrual disturbances were observed in almost 28.8% of patients with epilepsy, according to a study by Dr Bosak [11]. In contrast, another study found that a catamenial pattern exacerbation of seizures is present in almost 40% of women with epilepsy [14]. Studies as early as 1885 have recognized the correlation between seizures and menstruation. Further research contributed these findings to the pro-convulsant properties of estrogen as it predominantly hyperexcites neurons and lowers the seizure threshold. On the other hand, free progesterone and its metabolites demonstrated anticonvulsant properties, increasing the seizure threshold [10].

A study conducted in 1998 by Herzog and colleagues, involving 184 women with epilepsy, revealed that 78 patients (42.3%) exhibited one of the three patterns of catamenial epilepsy: C1-ovulatory cycles (35.7%), C2-premenstrual (28.5%), and C3-anovulatory (41.4%) [15]. The diagnosis of catamenial epilepsy is clinical. It is made after evaluating the seizure pattern and its relationship with specific phases of the menstrual cycle, noting an increase of more than two times in seizure frequency while excluding other potential causes of exacerbation. Seizure diaries are used to assess the occurrence of seizures during each phase of the menstrual cycle. Other ways to evaluate this relationship between menstrual cycle hormones and seizures include measuring serum progesterone levels (>3 ng/ml), which indicate the onset of ovulation. Additionally, changes in body temperature, where an increase of >0.7°F marks the start of the postovulatory phase, can also be used [12].

Several hypotheses exist detailing hormonal fluctuations and their impact on seizures, with the most accepted ones related to the cyclic variation in estrogen and progesterone levels during the menstrual cycle. The menstrual cycle consists of three main phases [16]. First, the follicular phase begins with menstruation and ends with ovulation. This phase is characterized by high estrogen and low progesterone levels, during which the dominant follicle matures and the endometrium thickens. Subsequently, the increasing estrogen levels trigger a positive feedback loop, releasing luteinizing hormone (LH) and the rupture of the mature follicle, releasing the ovum and starting the second phase called ovulation. Finally, the corpus luteum forms from the remnants of the dominant follicle and produces progesterone, which transforms the endometrium from a proliferative to a secretory phase. If fertilization does not occur, the corpus luteum degenerates into the corpus albicans, leading to a progressive decrease in progesterone and estrogen levels, which triggers menstruation [16].

This is important because we now understand the potential effects of estrogen and progesterone on the central nervous system. Estrogen, for example, has a pro-convulsant effect. It promotes the formation and density of synaptic spines, resulting in increased synapses, and at the same time regulates the expression of N-methyl-D-aspartate (NMDA) receptors, contributing to neuronal excitability [17]. Estrogen has a neuro-excitatory effect, which potentiates the neurons to respond to glutamate through presynaptic mechanism and lowers seizure threshold leading to CE [17]. It has positive feedback on NMDA-mediated receptors, leading to hyperexcitation and repetitive firing of hippocampal pyramidal neurons. This was supported by Smejkalova et al., who conducted a study on rats that concluded oestradiol exerts its effect on the hippocampus via the activation of NMDA receptors [17,18,19]. Furthermore, Studies demonstrated that estrogen could lead to the inhibition of GABA inhibitory effects in the amygdala and the substantia nigra which in turn promotes neuronal excitability and thus, the development of seizures in patients with epilepsy [20,21]. Lastly, it is worth noting that low estrogen exposure acts at beta-estrogen receptor, which has an anticonvulsant effect, increasing seizure threshold [22,23].

On the other hand, progesterone has demonstrated anticonvulsant effects in various animal models, possibly related to the potentiation of Gabaergic neurons. Progesterone can readily cross the blood-brain barrier, and it is believed that progesterone is a potent neuromodulator when converted into 3α-5α-THP (allopregnanolone) in the brain. 3α-5α-THP is a positive allosteric modulator that induces postsynaptic gamma-aminobutyric acid (GABA) neuro-inhibitory effect by increasing the duration chloride channels are open for like the mechanism of action of barbiturates but on different receptor sites, leading to a reduction in neuronal excitability and thus, increasing seizure threshold [10]. A study conducted on animals by Frye et al. determined that allopregnanolone does indeed have a neuroprotective effect on hippocampal neurons [22]. This protective effect is the highest in the mid-luteal stage when both progesterone and allopregnanolone are at peak concentration [24]. Other studies by Shiono et al. demonstrated that the induction of progesterone receptors in the limbic region led to a twofold increase in seizures in 57% of animals, highlighting the importance of free progesterone’s anti-convulsant properties [25,26]. Progesterone can also be converted into androgens and testosterone, both of these hormones demonstrated general antiseizure effects at higher doses [27]. It is worth noting that while progesterone and its metabolite are considered anticonvulsants, sulfated progesterone has pro-excitatory metabolites that suppress GABA [28,29]. 

Adrenal dehydroepiandrosterone sulfate (DHEA-S) is converted into oestradiol in the ovaries, while testosterone is converted into oestradiol in the hypothalamus and the limbic system [24]. Oestradiol is the most potent estrogen ligand-receptor and is usually elevated on the 10th day of the menstrual cycle. Androstenedione is converted into the least potent estrogen, oestrone. Although this hormone can also influence neuronal excitability, the low potency and the fact that this hormone is produced mainly by adipose tissue makes it the least likely form of estrogen to predispose epileptic patients to CE if they are not overweight. Lastly, oestriol is produced primarily by the placenta and thus this form of estrogen is elevated in pregnant women, though it can be made by the liver in smaller quantities through the hydroxylation of oestrone [30]. 

There are three patterns of catamenial seizures: C1 - premenstrual, which is related to “progesterone withdrawal,” occurring from day 25 to day 3 of the following cycle; C2 - periovulatory, associated with the abrupt rise in estrogen levels around days 10-15 of the cycle; and C3 - inadequate luteal phase, which occurs only in anovulatory cycles where, due to improper corpus luteum formation, progesterone levels are low while estrogen levels are preserved. This pattern occurs between days 10 and 3 of the following cycle [12].

Patients with C1-perimenstrual type are twice as likely to develop seizure exacerbation, as right before menstruation, progesterone receptors are induced, and progesterone is decreased, which in turn raises the risk of neuronal excitability [31]. The C2-periovulatory type occurs during the ovulatory stage between days 10 to 13 of the menstrual cycle. This is due to the isolated surge in estrogen levels, and thus its proconvulsant properties, which occur right before ovulation [32]. Lastly, the C3-luteal type occurs mostly in anovulatory women. Estrogen normally increases before ovulation; however, in these patients, the lack of ovulation leads to abnormally low levels of progesterone, which present as an increased estrogen: progesterone ratio and thus lower seizure threshold, provoking epileptic seizures [33]. As mentioned, progesterone can be produced by the corpus luteum if ovulation does not occur. The corpus luteum is not formed and hence, there is no progesterone production. This can result in a temporally diffuse manifestation of seizure during the second half of the anovulatory cycle [33,34]. 

During ovulatory cycles, the highest frequency of epileptic seizures is observed on day 1 of the cycle, corresponding with the drop in progesterone levels and a shift in the GABA-A receptor to a less sensitive form to benzodiazepines. Conversely, the lowest frequency of seizures occurs on day 8 of the menstrual cycle. This varies depending on the patient’s cycle length and the presence of anovulatory cycles [35]. For anovulatory cycles, another study by Herzog and colleagues evaluated a group of 92 women with clinically and electroencephalographically documented focal epilepsy over 3 months, comparing ovulatory cycles (progesterone >5 ng/ml) and anovulatory cycles. The result was a 29.5% increase in generalized tonic-clonic seizures during anovulatory cycles, with no impact on complex partial seizures or simple partial seizures. This suggests a link between anovulatory cycles and an increase in secondary generalized tonic-clonic seizures [36]. Table 1 compares estrogen and progesterone, focusing on their properties, effects on neuronal excitability, and impact on the seizure threshold.

**Table 1:** Comparison of estrogen and progesterone. NMDA receptors: N-methyl-D-aspartate receptors; GABA receptors: gamma amino butyric acid receptors. Table Credits: Hugh Kolomar

Feature	Estrogen	Progesterone
Predominant effect	Pro-convulsant	Anticonvulsant
Receptor interaction	Positive feedback on NMDA receptors	Positive modulation of GABA-A receptors
Impact on GABA	Inhibits GABAergic inhibition	Potentiates GABAergic inhibition
Impact on NMDA receptors	Upregulates NMDA receptor activity, leading to hyperexcitability	No significant direct activation of NMDA receptors
Net effect on seizure threshold	Lowers seizure threshold	Raises seizure threshold

Various treatments for epilepsy in women of reproductive age have been explored, including hormonal and non-hormonal therapies. Progesterone therapy, due to its anticonvulsant effects, has shown potential for managing catamenial epilepsy, though evidence remains inconsistent, and results vary depending on dosage and timing [12,32,37-39]. Gonadotropin-releasing hormone (GnRH) analogs, by suppressing pituitary hormones, may help in cases of irregular cycles but come with notable side effects like osteoporosis and cardiovascular risks [12]. Non-hormonal options include acetazolamide, clobazam, and lamotrigine, each showing varying degrees of efficacy in reducing seizure frequency, but more extensive studies are required to validate these findings [40-42]. Research on animal models has also highlighted compounds like ferulic acid and medicinal plants such as *Asparagus racemosus* as promising avenues for reducing seizure activity, though human applicability is yet to be determined [43-45]. A detailed discussion on the management of epilepsy in women of reproductive age, including these treatments, will be presented in a later section.

Pregnancy and epilepsy 

Like the menstrual cycle, hormones fluctuate throughout pregnancy. Consequently, pregnant women experience physical, physiological, and biochemical changes that impact every organ system [46]. Among the 50 million people affected by epilepsy globally, 1 in 5 are of childbearing potential [47]. This group is referred to as ‘people with epilepsy of childbearing potential’ (PWECP). Epilepsy in pregnancy is an area of research that has been advancing rapidly. Therefore, staying updated with emerging trends, guidelines, and research areas where potential gaps exist is crucial.

Since the 1980s, Ramsay has discussed the hypothesis that sex hormones significantly influence, if not drive, increased seizure activity during pregnancy [48]. Since then, more research has been conducted to understand this link with the attempt to fill research gaps.

When a woman becomes pregnant, fertilization and implantation occur, signaling the end of the luteal phase. The maternal-fetal-placental unit develops and ultimately becomes the primary producer of several sex hormones. This leads to a significant increase, far above the non-pregnant level, in circulating blood concentrations of neuroactive steroids (NAS), including sex steroid hormones and their metabolites [49-51]. NAS triggers accelerated nongenomic effects on neuronal excitability [49]. Generally, 17β-estradiol (EST), estradiol (E2), and estrogen exhibit pro-convulsant effects, whereas allopregnanolone (AP), 5α-tetrahydro deoxycorticosterone (THDOC), and progesterone (PROG) demonstrate anti-convulsant effects [49,52].

E2 is a biologically potent form of estrogen that has been shown to function as a post-transcriptional modulator, promoting increased neuronal excitability by exerting excitatory effects on N-methyl-D-aspartate receptors [52]. Despite estrogen’s role being mostly pro-convulsive in past literature, there has been suspicion of potential inhibitory effects. Li Q et al. reported that there have been proposals suggesting that estrogen, via a βE2 subtype, has the potential to exhibit anti-convulsive properties [52].

AP is a metabolite of PROG [53], which has been hypothesized to be involved in neurotransmitter synthesis and release. AP facilitates an increase in gamma-aminobutyric acid (GABA) neurotransmitters [52,53]. This results in the anti-convulsive properties of PWECP. Furthermore, there is suspicion of progestin’s ability to be involved in alternative mechanisms, such as influencing glutamate neurotransmission after binding to progesterone receptors, stimulating potential excitatory effects [52].

Other sex hormones, like androgen and testosterone, are typically much lower in females than estrogens, progesterone, and their metabolites. Regardless, exploring their influence in PWECP is still crucial. These hormones have been shown to demonstrate both anti- and pro-convulsant properties. Research from both animal and human studies indicates that they can convert into estrogens, potentially lowering the seizure threshold [52]. At the same time, they can also transform into neuro steroids and propanediol, which enhance GABAergic effects and help inhibit seizure activity [52].

Historical literature revealed that pregnancy did not alter the behavior of epileptic seizures [54]. Pennell et al. [55] multicenter cohort study found zero differences in seizure frequency between pregnant and non-pregnant women with epilepsy (WWE). Late in the twentieth century and, more recently, in the past few decades, numerous studies have been published demonstrating the concern between epilepsy and pregnancy with significant potential to worsen seizure frequency rates during pregnancy.

For instance, a more recent study demonstrated that an estimated 20% to 33% of pregnant WWE experienced an increase, 7% to 25% a decrease, and 50% to 83% no recordable change in seizure frequency [56]. Subsequent studies demonstrated similar patterns, thus investigating factors affecting seizure control during pregnancy. These factors include (a) patient noncompliance, (b) lower circulating concentrations of anti-epileptic drugs (AEDs) compared to dosages due to increased AED clearance, (c) higher levels of sex hormones, and (d) reluctance to use the most effective AED, valproate, which has potential teratogenic effects [53,54,56].

Voinescu PE et al. conducted a study on WWE at Brigham and Women’s Hospital between 2011 and 2019 [57]. The cohort size included 99 women and 114 pregnancies. The study identified two peaks: one in the first trimester and another near the end of the second trimester. It also suggested that a determinant of worsening seizure frequency during pregnancy involved the location of seizure onset. They found that women with focal epilepsy, particularly frontal epilepsy, had an increased risk of seizure worsening despite changes to therapeutic dosages. The study also reported that seizure frequency improved during the postpartum period, contradicting the hypothesis linking worsening control during pregnancy to sleep disturbances. Women with frontal lobe epilepsy often require polytherapy and remain refractory to AEDs, making seizure control challenging and posing risks to both the mother and fetus [57]. Therefore, large-scale studies are essential to validate earlier findings and include sleep studies to demonstrate a potential causality.

The complex interaction between pro-convulsive and anti-convulsive NAS is believed to influence seizure frequency during pregnancy [53]. It has also been suggested that NAS can also affect seizure control [53]. There are also conflicting reviews of the literature potentially examining whether human chorionic gonadotropin (hCG) plays any role in increasing or decreasing seizure frequency during pregnancy.

The European Registry of ASD and Pregnancy (EURAP) study is an observational, longitudinal, cohort study spanning 47 countries and involving more than 1,500 collaborators with a timeline from 1999 to 2022 [58]. EURAP demonstrated an increase in the number of seizures during pregnancy; however, contributing factors mainly focused on AEDs. Currently, no clearly defined mechanisms demonstrate how NAS contributes to increased or decreased seizure frequencies during different trimesters in pregnancy in the collated studies. There are rather hypotheses about potential mechanisms. For example, the exact influence of elevated estrogen levels, known to be pro-convulsive, is not as clear during pregnancy [53]. Consequently, this sparks an area that requires further investigation.

Epilepsy and pregnancy outcomes

A recent study by Kuang H et al. [59], a systematic review and meta-analysis, examined maternal and fetal outcomes among pregnant WWE. Higher risks were observed, including cesarean section, hypertensive disorders, preeclampsia/eclampsia, induction of labor, antepartum hemorrhage, postpartum hemorrhage, diabetes mellitus, vaginal bleeding, congenital malformations, fetal distress, small for gestational age, spontaneous abortion, preterm birth, fetal growth restriction, and low birth weight (<2500 g) [59-61].

Most women have uncomplicated pregnancies, but they are considered a higher risk due to increased mortality rates [59]. The revised practice guidelines (2024) from the American Academy of Neurology (AAN), the American Epilepsy Society (AES), and the Society for Maternal-Fetal Medicine (SMFM) indicate that the mortality risk for pregnant WWE is 5 to 12 times greater [47]. While it is true many of the risks associated with pregnant WWE are like those faced by non-pregnant WWE [59], further caution is needed to combat complications and harm to both mom and fetus.

Many recent studies demonstrated a moderate increase in gestational hypertensive disorders in WWE. However, earlier research demonstrated no significant variations in preeclampsia rates in WWE compared to the control cohort [59]. National registries in Sweden, the United States, and Norway highlighted slightly higher rates of preeclampsia in WWE compared to controls. Other risk factors included cardiovascular factors and AED use, where outcomes varied by medication type and primiparity [59,60]. A recent study indicated that the monotherapy use of lamotrigine and levetiracetam, newer AEDs, did not predispose to preeclampsia [60]. These studies suggest that there is no single causal link to hypertensive disorders among pregnant WWE; rather, they indicate a mechanism involving both epilepsy and a combination of AEDs.

There has been an increase in the rate of cesarean deliveries in WWE, having doubled compared to the general population, indicating a notable shift in the preferred delivery method [59]. Additionally, there has been a similar risk associated with the induction of labor and spontaneous abortion during pregnancy WWE [60]. However, C-sections come with their risks and complications, and hence, epilepsy alone does not warrant this shift to C-sections unless this is in the best interest of the mother and fetus [59,60]. Post-partum hemorrhage is also increased in WWE, with high rates contributing to those underdoing c-sections [60]. More studies are indicated for hemorrhage risks associated with vaginal deliveries in pregnant WWE. No significant change was noted in those who had forceps or vacuum-assisted deliveries [60].

Adverse fetal outcomes in pregnant WWE remain poorly understood, often linked to seizure-related trauma and AED use [62]. Some studies suggest that epilepsy pathology, rather than AEDs, contributes to maternal and fetal outcomes. Trauma during pregnancy can lead to fetal membrane rupture, premature delivery, and infection risk [59]. Pregnant WWE faces higher risks of intrauterine growth restriction and congenital malformations compared to non-pregnant WWE, influenced by factors such as AEDs, seizure trauma, genetics, environment, and pre-existing conditions [59]. Premature or low-birth-weight neonates are at increased risk of complications and illnesses later in life, highlighting the need for detailed research to optimize seizure management. Furthermore, levetiracetam was also linked to neurodevelopmental and neuropsychiatric risks, including ADHD and anxiety, with emerging concerns about topiramate’s neurodevelopmental effects (Table 2) [59].

**Table 2:** Maternal and fetal complications in women with epilepsy. AED: anti-epileptic drugs; ADHD: attention deficit hyperactivity disorder. Table credits: Hugh Kolomar.

Complication	Maternal risk factors	Fetal risk factors
Cesarean section	Seizure frequency, AED use	Obstructed labor, epilepsy-related complications
Hypertensive disorders (e.g., preeclampsia)	AED use, cardiovascular comorbidities, epilepsy pathology	Placental dysfunction, AED exposure
Antepartum hemorrhage	Seizure-related trauma, AED use	Placental abruption due to seizure trauma
Postpartum hemorrhage	Higher in C-section deliveries, AED use	Birth-related trauma, complications of C-section
Preterm birth	Seizure-related trauma, AED use	Maternal seizures, placental insufficiency
Low birth weight (<2500g)	Epilepsy pathology, seizure-related trauma	Placental insufficiency, maternal malnutrition
Congenital malformations	AED exposure, particularly valproate and polytherapy	Teratogenic effects of AEDs, particularly older AEDs
Fetal growth restriction	AED use, epilepsy pathology	Placental insufficiency, AED use
Neurodevelopmental disorders (e.g., ADHD, autism)	AED exposure, particularly valproate, topiramate	In utero exposure to neurotoxic AEDs

Management strategies for epilepsy during pregnancy

Over the last five decades, concerns have arisen regarding the risk of congenital malformations linked to exposure to AEDs prenatally and during pregnancy [63]. The main reason for treating pregnant WWE using AEDs is to achieve seizure control and prevent related adverse effects, including complications for both mother and fetus, as discussed above [59]. To tackle these issues, independent research groups designed and implemented prospective registries to evaluate the risk of major congenital malformations (MCMs) resulting from maternal AED use [63]. Registries include the European registry of antiepileptic drugs and pregnancy (EURAP), the North American antiepileptic drug pregnancy registry, the UK and Ireland epilepsy and pregnancy registries, and the Nordic registry, which aim to identify the risks associated with AED use during pregnancy, thereby informing management decisions. These have been discussed in the current literature and the 2024 updated guidelines by AAN, AES, and SMFM [59]. 

Global AED use among pregnant WWE has nearly tripled in the past 12 years [63]. Physiological changes during pregnancy significantly alter AED pharmacokinetics, requiring dosage optimization to balance seizure control and minimize adverse effects [53]. Increased water content and fat reserves expand drug distribution volumes, reducing serum concentrations. Enhanced activity of cytochrome P450 enzymes and UGTs during pregnancy accelerates AED metabolism, reducing drug serum levels. Lower albumin levels also increase the active, unbound drug fraction. Increased renal clearance further reduces AED levels due to heightened blood flow and glomerular filtration rates [53]. Additionally, AEDs cross the placenta, potentially disrupting fetal development and posing teratogenic risks.

Effective seizure management during pregnancy necessitates monitoring and adjusting AED dosages to maintain therapeutic levels. The American Academy of Neurology (AAN) updated guidelines to help manage pregnant WWE, reducing risks of neurodevelopmental, perinatal, and congenital outcomes [47]. A shift from older to newer AEDs has emerged for improved pregnancy and postpartum management, although data on newer AEDs remain limited [47]. AED dosage adjustments are recommended for reduced serum levels or worsening seizure control, often due to increased drug clearance rather than reduced bioavailability [64].

First-Generation AEDs

Valproate is discouraged in pregnancy due to teratogenic risks, with its clearance increasing throughout pregnancy, necessitating dosage adjustments [53,58]. Phenobarbital, primidone, phenytoin, oxcarbazepine, and carbamazepine require dose changes due to enhanced metabolism, particularly in the third trimester [53,64]. Carbamazepine can induce its metabolism with exaggerated changes in late pregnancy. Oxcarbazepine shows one of the lowest serum levels and has a short half-life, further complicating management [64].

Second-Generation AEDs

Levetiracetam clearance increases significantly in the third trimester, requiring careful monitoring to avoid seizures. Lamotrigine metabolism rises in late pregnancy due to UGT enzyme upregulation, with low serum levels reported. Topiramate and zonisamide require dose adjustments due to increased renal clearance and hepatic metabolism, particularly in the third trimester [53,64]. 

Third-Generation AEDs

Perampanel clearance increases due to upregulated hepatic and UGT enzymes, necessitating higher doses [64]. Data on third-generation AEDs remain limited, with adjustments typically based on clinician discretion due to small cohort studies and lack of robust evidence [47]. 

Teratogenic risks

It is well known that teratogenic effects are common among older AEDs like valproate [65]. The EURAP, the North American antiepileptic drug pregnancy registry, and the UK and Ireland epilepsy and pregnancy registries highlighted teratogenic risks associated with varying AEDs and dosages, with the highest risks of MCMs in valproate, phenobarbital, and phenytoin, respectively, in comparison to more recent AEDs like lamotrigine, carbamazepine, oxcarbazepine and levetiracetam [47,56]. Polytherapy use of AEDs during pregnancy has been shown to have worse teratogenic risks and MCMs when compared to monotherapy. This is hypothesized to be due to cumulative and synergistic drug effects but can also be due to the addition of valproate and topiramate in the regimen [53]. Cardiac malformations were identified as the number one cause of anomalies/MCMs across all AEDs [63]. Exposure to AED topiramate during pregnancy has been associated with cleft lip or palate [53]. Valproate exposure has been linked to various malformations in many organ systems, causing cardiac anomalies, nervous system anomalies, oral clefts, hypospadias, clubfoot, etc. [47,53]. Interestingly, the Nordic registry demonstrated that MCMs associated with valproate and topiramate were dose-dependent [53]. 

The neurodevelopmental effects of anti-seizure drug (ASD (NEAD)) study revealed a reduction in IQ scores, and another observational study revealed a decline in performance across six neurocognitive domains, especially language, in children exposed to valproate compared to those exposed to other AEDs [53]. The EURAP study revealed that during the first trimester, pregnant WWE who experienced tonic-clonic seizures did not show any association with MCMs [63]. Xu SC et al., discussed the potential links between AEDs and autism spectrum disorder and/or attention deficit hyperactivity disorder [62]. However, studies have been inconsistent. AEDs affect the dopaminergic system in the fetus, disrupting neurodevelopment as they pass from the mother through the placenta to the fetus [47]. 

Antiepileptic drugs and reproductive health

Antiepileptics (AEDs) are mainly of two types: one inducing enzyme (EIAED'S) such as phenytoin (PHT), carbamazepine (CBZ), oxcarbazepine (OXC), and phenobarbital (PB) and one that doesn't induce (NEIAEDs). EIAED exerts influence on cytochrome P450 (CYP) from the liver. These increase sex hormone-binding globulin (SHBG) levels and lower endogenous sex steroid concentrations gradually [9]. Gabapentin or lamotrigine, the NEIAEDs resulted in no change in sex steroid hormone levels when treated in women with epilepsy [9].

Valproate (VPA), being one of the most frequently used AED [9], impedes the conversion of testosterone to estradiol by aromatase and testosterone to estrogen by epoxide hydrolase respectively [66], leading to hormonal disbalance such as hyperandrogenemia and hyperinsulinemia [9], as well as broken menstrual cycle, polycystic ovaries, and infertility in women [66]. Such changes may explicitly and implicitly influence weight gain, causing infertility [9]. Half the fraction of cycles in women are anovulatory when treated with VPA [9]. 

Women with epilepsy tend to have more frequent polycystic ovary syndrome (PCOS) compared to healthy ones. Major research established that women tend to have a broken menstrual cycle in epilepsy compared to valproate, carbamazepine, and other AEDs. The PCO morphology and testosterone were increased in women consuming valporate than non-valproate [67]. The effects of GnRH mediated by GABA in the central nervous system and the indirect weight gain, which results in insulin resistance, are two ways that valproate may cause PCOS symptoms [67]. Women commencing on valproate monotherapy or adjuvant therapy lately are highly prone to PCOS and are exposed to PCOS traits within a few months [9].

Some metabolic abnormalities, ovarian dysfunction, and hypothalamic-pituitary axis malfunction may all result in ovulatory failure. Researchers proposed women may encounter unusual pituitary responses to infused LHRH in temporal lobe epilepsy. In addition, women using AEDs may experience abnormalities in LH, FSH concentrations, and pulsatile LH release [68].

When lamotrigine (LTG) is given instead of VPA, the hyperandrogenism and insulin resistance can be reverted [9]. LTG does not modify metabolic, endocrine functions, or body weight [69]. PB has a shielding effect by lowering biologically activated testosterone and elevating its binding and metabolism against the growth of PCOS [70]. Eventually, it does not remain very certain if VPA directly contributes to the genesis of PCOS. Currently, endocrine and biochemical changes resulting in PCOS can't be linked to body weight gain [70]. VPA remains the treatment of choice in epilepsy, especially in young women. It is always important to track the menstrual cycle length and body weight of women initiating VPA therapy, as VPA is always linked to endocrine and reproductive issues rather than other AEDs [70].

Epilepsy surgery 

Epilepsy surgery is used to manage seizures when drugs fail. While the primary goal is seizure reduction, it is critical to examine the potential consequences on reproductive health. According to research, successful epilepsy surgery can improve health-related quality of life (HRQOL), including reproductive health. A multicenter cohort study of 265 patients found that surgical patients' HRQOL improved during the first year after surgery [71]. 

Temporal lobe epilepsy is most commonly caused by focal epilepsy. Surgery for temporal lobe epilepsy can control seizures and improve the quality of life in appropriately selected patients. However, 20-30% of patients do not respond to medical or surgical treatment. Managing chronic intractable epilepsy requires comprehensive care to address events of medical treatment, quality of life issues, and comorbid diseases [72].

Other treatment options 

For individuals opting for non-surgical management of epilepsy, lifestyle modifications and hormonal interventions can play a crucial role in both seizure control and reproductive health [73]. Lifestyle changes adopting a healthy lifestyle is crucial for individuals with epilepsy, as it can enhance overall well-being and potentially improve seizure control. Key lifestyle considerations include: nutrients highlights that patients with epilepsy often have dietary inadequacies, consuming less fruit, pulses, nuts, and seeds, and more sugar-sweetened beverages. Addressing these dietary habits is essential for overall health and may influence seizure control [73]. Exercise training can improve fitness, psychosocial, and neurocognitive results in patients with epilepsy. Overall, the data indicates that patients with epilepsy can benefit from exercise [74].

Fluctuations in serum progesterone and estrogen levels during a normal reproductive cycle enhance or reduce the chance of seizure development, depending on the serum estradiol/progesterone ratio. As a result, progesterone, its metabolite allopregnanolone, and other hormone treatments have been investigated in the treatment of people with epilepsy [75].

Contraception and preconception counseling 

Effective contraception is especially necessary for women with epilepsy who are of reproductive age due to antiepileptic drug-related teratogenicity and hormonal interactions, nevertheless, studies show that many women do not obtain contraceptive and periconceptional counseling. Management concerns in this population include a high risk of pregnancy complications and peripartum psychological issues compared to women without epilepsy. To maximize therapy of poor bone density in women with epilepsy, investigations exploring bone densitometry frequency and calcium and vitamin D supplementation are necessary [75].

It was created in response to early concerns about the potentially increased risk of congenital malformation in infants born to mothers with epilepsy, as well as recognition of the need to provide both education and counseling on aspects [76].

Preconception counseling is the process of planning and preparing for pregnancy to optimize physical, mental, and emotional health before conception, therapy reducing poor pregnancy outcomes and enhancing the long-term health of both mother and child. Counseling interventions are limited to promoting ongoing review of epilepsy management, encouraging the use of contraception to delay pregnancy, and ensuring that patients can conceive in optimal health with the fewest risk factors, are fully aware of any risks and benefits of treatment, and can make informed decisions about future pregnancies [76]. Preconception counseling is a targeted educational and counseling intervention that is best delivered before conception and consists of three major components: risk assessment, health promotion, and intervention [76].

Women with epilepsy planning to conceive should take folic acid at a dose of 5 mg/day during the preconception period and throughout pregnancy. For women on enzyme-inducing antiepileptic drugs (AEDs), vitamin K supplementation is recommended during the final month of pregnancy. The use of phenytoin, valproate, carbamazepine, lamotrigine, and phenobarbitone has been linked to an increased risk of major congenital malformations and minor morphological abnormalities [77]. While valproate may be the most appropriate option for certain women with epilepsy, its risks and benefits should be carefully evaluated and discussed with the patient [77]. Other therapeutic options, such as vagal nerve stimulation, responsive neurostimulation, and deep brain stimulation, are generally considered safe [78].

Vaginal delivery is typically recommended. A cesarean delivery may be indicated in cases where poor seizure control during pregnancy or a high risk of seizures during labor could compromise the delivery process and increase the risk of complications. Epidural anesthesia is also recommended, as is the use of prostaglandins for labor induction [37].

In summary, planned pregnancies are associated with better seizure control and reduced fetal exposure to AEDs during pregnancy. In contrast, unplanned pregnancies often necessitate immediate adjustments to AED doses upon confirmation of pregnancy, which can result in a higher incidence of seizures during gestation. Nevertheless, planned pregnancies are shown to improve maternal and neonatal outcomes during the peripartum period [79].

Research gaps and future directions

Reproductive hormones play a key role in the pathophysiology of seizures, with estrogen often exerting proconvulsant effects and progesterone providing anticonvulsant protection. Fluctuations in the estradiol/progesterone ratio significantly influence seizure risk, particularly in women with catamenial epilepsy. While treatments like allopregnanolone and hormone replacement therapy (HRT) have shown promise, their efficacy and safety require further research, as HRT may exacerbate seizures depending on its formulation [75,80-82]. Estrogen’s influence extends beyond reproductive functions, affecting various brain regions linked to cognition, motor coordination, and emotion, though its exact mechanisms remain unclear [81].

Managing epilepsy during pregnancy poses challenges due to risks of teratogenicity and seizure exacerbation. Preconception planning, including selecting low-risk anti-seizure medications (ASMs), high-dose folic acid supplementation, and collaborative neuro-obstetric care, is critical for optimizing outcomes. Registries have provided valuable insights into medication effects on fetal development, yet gaps in understanding remain [7,37,83]. Long-term studies are needed to refine treatment protocols and ensure safety, particularly for women planning pregnancies or managing catamenial epilepsy, where therapeutic options remain limited [7,83,84-86].

## Conclusions

Hormonal changes significantly influence epilepsy, with fluctuations during the menstrual cycle often exacerbating seizure activity, especially in catamenial epilepsy. These changes underscore the critical need for a personalized, multidisciplinary approach to epilepsy management that integrates biological, psychological, and social factors. Tailored care strategies should address the specific needs of women during different life stages, including the menstrual cycle, pregnancy, and postpartum period, to optimize seizure control and overall well-being. Collaborative efforts among neurologists, gynecologists, endocrinologists, and mental health professionals are essential to achieving these goals. By adopting individualized treatment plans that consider patient preferences and life circumstances, healthcare providers can significantly improve quality of life, reduce seizure-related complications, and empower women to manage their conditions effectively.
